# Usefulness of Blood–Urea–Nitrogen to Serum Albumin Ratio for In-hospital Mortality Predictions in Atrial Fibrillation Patients Admitted to the Intensive Care Unit: A Retrospective Analysis From MIMIC-IV Database

**DOI:** 10.31083/RCM36596

**Published:** 2025-07-29

**Authors:** Han Xie, Qing Luo, Ting Huang

**Affiliations:** ^1^Department of Cardiology, The Central Hospital of Wuhan, Tongji Medical College, Huazhong University of Science and Technology, 430014 Wuhan, Hubei, China; ^2^Key Laboratory for Molecular Diagnosis of Hubei Province, The Central Hospital of Wuhan, Tongji Medical College, Huazhong University of Science and Technology, 430014 Wuhan, Hubei, China

**Keywords:** blood urea nitrogen, serum albumin, atrial fibrillation, intensive care unit, hospital mortality, nomogram

## Abstract

**Background:**

Despite prior research showing that elevated BAR levels were linked to poor prognoses in several cardiovascular disease conditions, the predictive role of the blood–urea–nitrogen to serum albumin ratio (BAR) in atrial fibrillation (AF) patients admitted to the intensive care unit (ICU) remains largely unknown.

**Methods:**

Patients diagnosed with AF were gathered from the Medical Information Mart for Intensive Care-IV (MIMIC-IV) database, and the X-tile software was used to determine the best cut-off values for BAR. The Kaplan–Meier curves and receiver operating characteristics (ROC) analyses were used to evaluate the prognostic value of the BAR. The identified prognostic indicators were used to build a nomogram model.

**Results:**

Finally, 13,451 AF patients were included in this study. The best BAR cut-off value was 8.9. In-hospital survival was substantially higher in the low-BAR group (BAR ≤8.9) than in the high-BAR group (BAR >8.9) (HR: 3.15, 95% CI: 2.89–3.44; *p* < 0.001). A nomogram model was developed using the findings of multivariable logistic regression, considering variables such as age, heart rate, albumin, white blood cell count, simplified acute physiology score II (SAPS II) score, sequential organ failure assessment (SOFA) score, mechanical ventilation, and the BAR. When forecasting the probability of death for AF patients admitted to the ICU, the nomogram showed good performance and practical application. Calibration curves evaluated the accuracy of the model, decision curve analysis evaluated the clinical use of the model, and the area under the receiver operating characteristic (AUROC) curve evaluated the discriminative capabilities of the model.

**Conclusion:**

Among critically ill AF patients, the BAR, a readily available clinical measure, shows outstanding predictive ability in predicting in-hospital mortality. Additionally, in-hospital mortality could be predicted with high accuracy using a nomogram that included the BAR.

## 1. Introduction

Cardiovascular diseases (CVD) have been the world’s leading cause of death in recent decades. According to reports, cardiovascular diseases claimed the lives of almost 20.5 million individuals in 2021, making up about one-third of all fatalities worldwide [1]. Among these prevalent cardiovascular diseases, atrial fibrillation (AF) is significantly associated with an increased risk of all-cause mortality, heart failure, hospitalization, and thromboembolic events [2, 3]. AF is notably prevalent among critically ill patients, representing approximately 47.4% to 61% of all arrhythmias and about 52% of atrial arrhythmias in intensive care units (ICUs), highlighting its significance in this setting [4, 5]. AF is also linked to poor prognosis, as evidenced by the atrial fibrillation (AFIB)-ICU cohort study, which found that patients with AF had a 1.38-fold higher risk of 90-day mortality compared to those without AF [6]. Another study underscored that both new-onset and recurrent AF in ICU significantly increased mortality among hospitalized patients [7]. The French and euRopean Outcome reGistry in Intensive Care Unit (FROG-ICU) research further revealed that patients with new-onset AF had approximately a 60% higher risk of in-hospital mortality compared to those without AF [8]. Consequently, there is a pressing need to develop a novel predictive model to identify patients at high risk of mortality.

Mostly eliminated by the kidneys, blood urea nitrogen (BUN) is the main byproduct of human protein metabolism. Increased BUN levels are a critical indicator of a patient’s renal function and protein catabolism status and arise in situations of either impaired glomerular filtration rate or excessive protein catabolism [9]. Similarly, albumin has a number of physiological characteristics, including as anti-inflammatory and antioxidant actions, and indicates the body’s nutritional state. A composite metric with important therapeutic consequences, the BUN/albumin ratio (BAR) can concurrently represent the body’s nutritional state, inflammation, and liver and kidney function [10]. BAR has shown significant value in a number of acute and severe illnesses, enabling the evaluation of disease severity and prognostication prediction. Previous research has demonstrated BAR’s exceptional prognostic prediction power in a wide range of illnesses, such as sepsis, pneumonia, acute renal insufficiency, chronic heart failure, and chronic obstructive pulmonary disease [11, 12, 13, 14].

However, the unique pathophysiological characteristics of AF patients in the ICU—such as hemodynamic instability, heightened inflammatory responses, and electrolyte imbalances—may influence the prognostic utility of BAR differently compared to other critically ill populations. Therefore, it is imperative to investigate the specific role of BAR in predicting in-hospital mortality among ICU patients with AF. This study aims to explore this potential, with the goal of enhancing clinical assessment and guiding individualized treatment strategies for this high-risk group.

## 2. Methods

### 2.1 Data Source

This retrospective observational study utilized data from the Medical Information Mart for Intensive Care IV (MIMIC-IV) database, maintained by the Massachusetts Institute of Technology’s Laboratory for Computational Physiology. The database comprises de-identified health records of patients admitted to the intensive care units at Beth Israel Deaconess Medical Center (BIDMC) in Boston between 2008 and 2019. The Institutional Review Board of BIDMC approved the use of this dataset for research purposes and waived the requirement for informed consent due to the anonymized nature of the data. The author (HX) acquired the required permissions and met all database access restrictions.

### 2.2 Cohort Selection

**Fig. 1.**
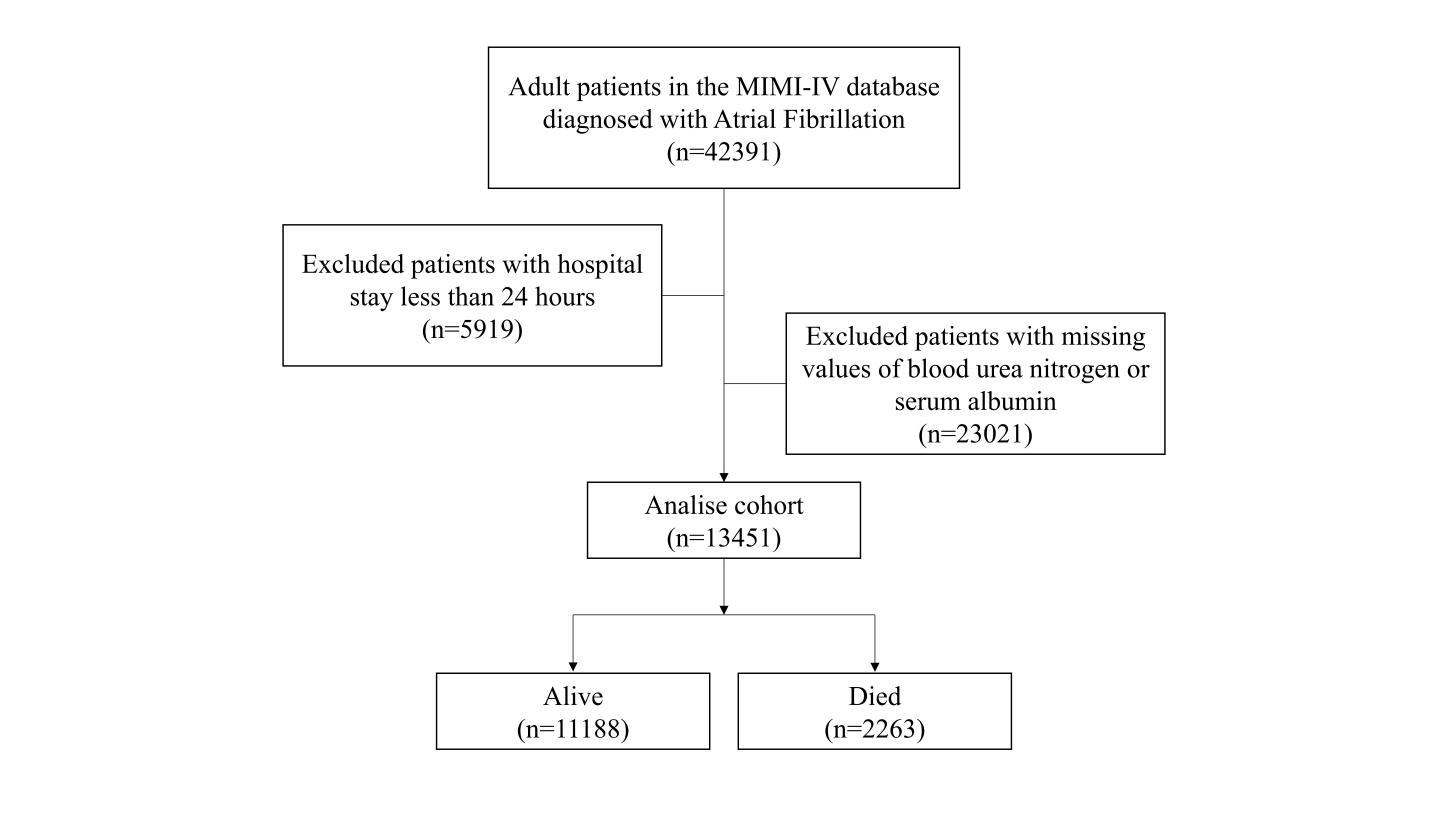
**The flow chart of this study**. MIMIC-IV, Medical Information Mart for Intensive Care IV.

Patients diagnosed with AF were identified using International Classification of Diseases, Ninth Revision, Clinical Modification (ICD-9-CM) code 427.31. Only the first hospitalization record for each patient was included. Patients those with hospital stays shorter than 24 hours, and those with missing serum urea nitrogen or albumin measurements within the first 24 hours of admission were excluded from the analysis. Finally, the analysis encompassed 13,451 AF patients (Fig. 1).

### 2.3 Data Collection

Baseline demographics (age, sex, height, weight), comorbidities (myocardial infarction, heart failure, diabetes mellitus, chronic renal disease defined by ICD-9 codes), 24-hour ICU admission laboratory data, and admission severity scores (Oxford Acute Severity of Illness [OASIS] [15], simplified acute physiology score II [SAPS II] [16], sequential organ failure assessment [SOFA] [17]) were extracted from the MIMIC-IV database via PostgreSQL version 9.6 (The PostgreSQL Global Development Group, San Francisco, CA, USA) using Structured Query Language (SQL).

### 2.4 Statistical Analysis

The statistical studies were conducted using R software version 4.1.2 (University of Auckland, Auckland, New Zealand), X-tile (Yale University, New Haven, CT, USA), and SPSS 22.0 (IBM Corp., Armonk, NY, USA). For baseline comparisons, the patients were split into high- and low-BAR groups according to the ideal cut-off value for BAR, which was determined using X-tile software (​In this study, we utilized X-tile software to determine the optimal cutoff value for the BAR. X-tile evaluates all potential cutoff points by systematically assessing the association between BAR and patient outcomes, employing Kaplan-Meier survival analysis and log-rank tests to identify the threshold that best stratifies patients with significant survival differences. This approach enables an objective determination of the most informative cutoff value for subsequent analyses). Use the normality test to examine the normality of continuous variables Normally distributed variables were expressed as mean ± standard deviation (SD) and compared using the independent-samples *t*-test. Non-normally distributed variables were expressed as median (interquartile range, IQR) and compared using the Wilcoxon rank-sum test. Categorical variables were presented as counts (percentages) and compared using Fisher’s exact test or the chi-square test, as appropriate. High- and low-BAR groups’ in-hospital survival rates were compared using Kaplan-Meier survival curves, and any differences were evaluated using the log-rank test. To find risk factors linked to in-hospital mortality among critically unwell AF patients, least absolute shrinkage and selection operator (LASSO) regression was used. Multivariate logistic regression was then used for additional refinement. Subsequently, a nomogram was developed to visually represent the risk prediction model. ​In this study, we conducted internal validation of the nomogram model using the R programming language and the rms package. We constructed a logistic regression model with the lrm() function and defined the data distribution using datadist(). To assess model calibration, we applied the calibrate() function with 1000 bootstrap resamples (method = “boot”, B = 1000), generating bias-corrected calibration curves. The plot() function was then used to visualize the agreement between predicted probabilities and observed outcomes. This approach provides an optimism-corrected evaluation of the model’s predictive performance. The area under the receiver operating characteristic (ROC) curve was used to measure the model’s discriminative power, accuracy was measured using calibration curves, and the model’s clinical utility was tested by decision curve analysis. To assess the model’s resilience in various situations, univariate and multivariate Cox regression analyses were also performed. Two-sided *p*-values below 0.05 were considered statistically significant.

## 3. Results

### 3.1 Patient Characteristics

**Fig. 2.**
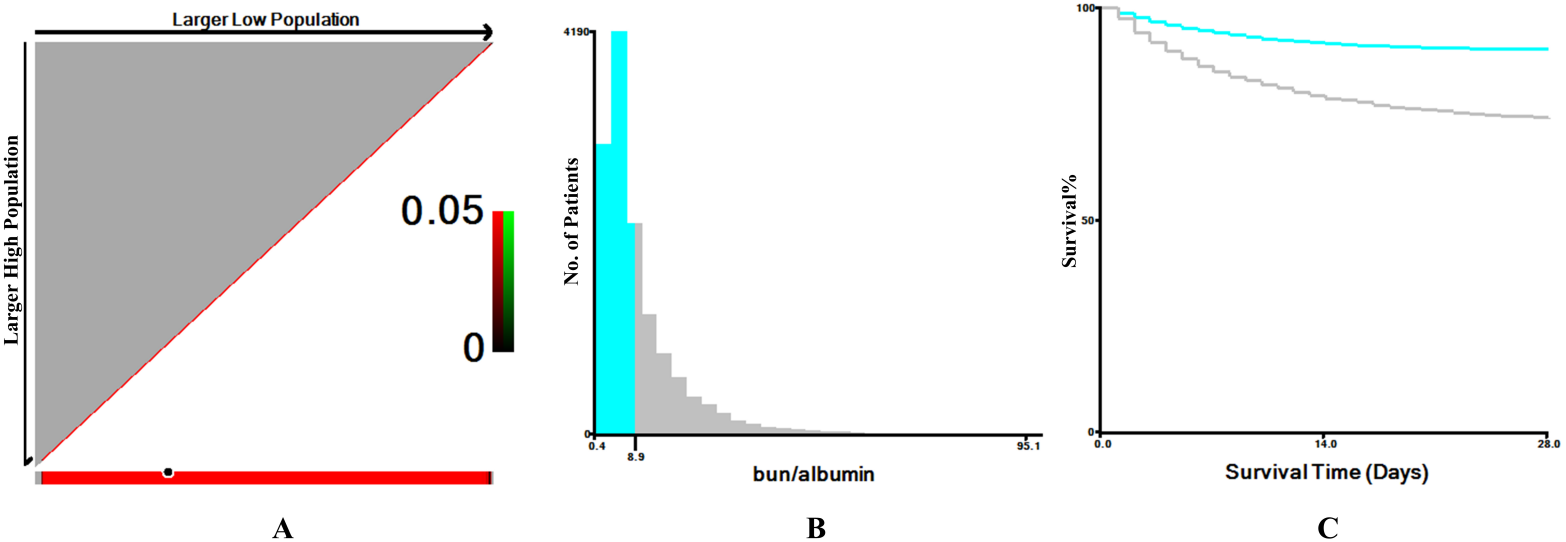
**The optimal cutoff value for the blood urea nitrogen to serum albumin ratio (BAR) was determined using X-tile software**. (A) In the X-tile plots (left panels), the optimal cutoff point is indicated by a black circle. (B) The middle panels display histograms illustrating the distribution of BAR values and the selected cutoff. (C) The right panels present Kaplan-Meier survival curves, demonstrating the prognostic significance of the identified cutoff in critically ill patients with atrial fibrillation.

Ultimately, 13,451 critically ill AF were collected, among which 2263 (16.82%) experienced in-hospital mortality, as shown in Fig. 1. The optimal cut-off value for BAR was 8.9. Following that, patients were split into two groups according to the ideal cut-off value, as shown in Fig. 2. In this study, 13,451 AF patients were categorized into low-BAR (≤8.9, n = 8672) and high-BAR (>8.9, n = 4779) groups based on the optimal cut-off value of the BAR. Compared to the low-BAR group, the high-BAR group exhibited older age, higher male proportion, and increased body weight (all *p* < 0.001). They also had higher incidences of myocardial infarction, congestive heart failure, diabetes, and chronic kidney disease (all *p* < 0.001). Intervention rates, including mechanical ventilation and vasopressor use, were significantly elevated in the high-BAR group (*p* < 0.001). Severity scores such as SOFA, OASIS, and SAPS II were notably higher (all *p* < 0.001). Laboratory findings indicated elevated white blood cell counts, blood urea nitrogen, creatinine, and BAR values, alongside decreased hemoglobin, platelet counts, and albumin levels (all *p* < 0.001). Clinically, the high-BAR group experienced longer ICU and hospital stays and a higher in-hospital mortality rate (27.82% vs. 10.75%, *p* < 0.001). These results suggest that a higher BAR is associated with more severe baseline conditions and poorer outcomes in critically ill AF patients, Table 1 displays the baseline attributes.

**Table 1.** **The baseline characteristics of all patients**.

Characteristics		Low-BAR (≤8.9)	High-BAR (>8.9)	*p* values
		N = 8672	N = 4779	
Age, years old		61 ± 18	68 ± 15	<0.001
Gender, male, n (%)		4680 (53.97)	2856 (59.76)	<0.001
Weight, kg		80 ± 23	84 ± 26	<0.001
Comorbidities, n (%)				
	Myocardial infarct	1228 (14.16)	1013 (21.20)	<0.001
	Congestive heart failure	1792 (20.66)	1940 (40.59)	<0.001
	Diabetes	2026 (23.36)	1851 (38.73)	<0.001
	Chronic kidney disease	127 (1.46)	566 (11.84)	<0.001
	Charlson comorbidity index	5 (3, 7)	5 (4, 7)	0.467
Interventions, n (%)				
	Mechanical ventilation	1208 (13.93)	953 (19.94)	<0.001
	Vasopressors	2462 (28.39)	2232 (46.70)	<0.001
Score system, points				
	SOFA	4 (2, 6)	8 (5, 11)	<0.001
	OASIS	32 ± 9	37 ± 10	<0.001
	SAPS II	31 (24, 40)	46 (37, 56)	<0.001
Vital signs				
	Mean arterial pressure, mmHg	81 ± 12	75 ± 11	<0.001
	Heart rate, bpm	86 ± 17	88 ± 17	<0.001
Laboratory values				
	White blood cell, ×10^9^/L	10.50 (7.65, 14.15)	11.75 (8.15, 16.70)	<0.001
	Hemoglobin, g/dL	11.45 ± 2.11	10.03 ± 1.99	<0.001
	Platelet, ×10^9^/L	199 (145, 259)	174 (113, 246)	<0.001
	Albumin, g/dL	3.47 ± 0.65	2.95 ± 0.65	<0.001
	Nitrogen	15.3 (11.3, 20.5)	44.3 (34.0, 62.2)	<0.001
	BAR	4.5 (3.2, 6.3)	14.9 (11.3, 21.7)	<0.001
	Creatinine, mg/dL	0.85 (0.68, 1.10)	1.90 (1.30, 3.13)	<0.001
	Bicarbonate	23.17 ± 4.01	21.03 ± 5.01	<0.001
Outcomes				
	Length of ICU stay	2 (2, 5)	3 (2, 6)	<0.001
	Length of hospital stay	7 (4, 12)	9 (5, 17)	<0.001
	In-hospital death, n (%)	932 (10.75)	1331 (27.82)	<0.001

SOFA, sequential organ failure assessment; OASIS, Oxford Acute Severity of Illness Score; ICU, intensive care unit; SAPS II, simplified acute physiology score II; BAR, blood urea nitrogen albumin ratio.

### 3.2 BAR Is an Independent Risk Factor for In-hospital Mortality in ICU Patients With AF

**Fig. 3.**
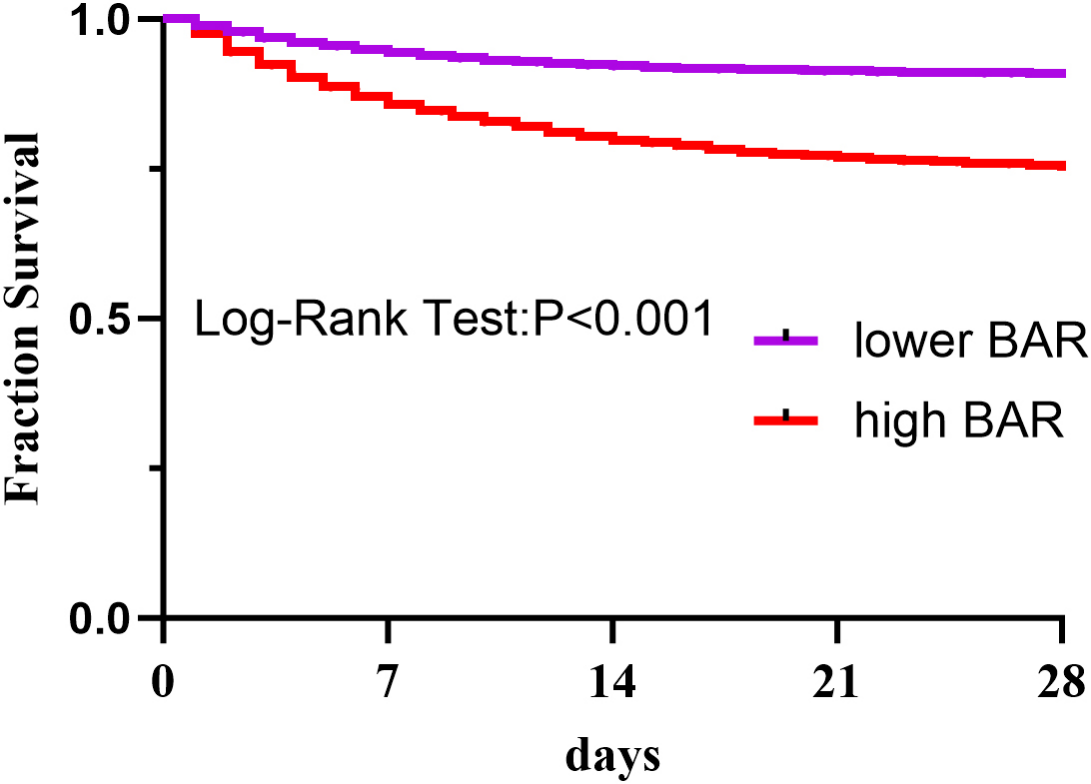
**The Kaplan-Meier curve was used to determine the overall survival of the high-BAR and low-BAR groups**. BAR is the ratio of blood urea nitrogen to serum albumin.

**Fig. 4.**
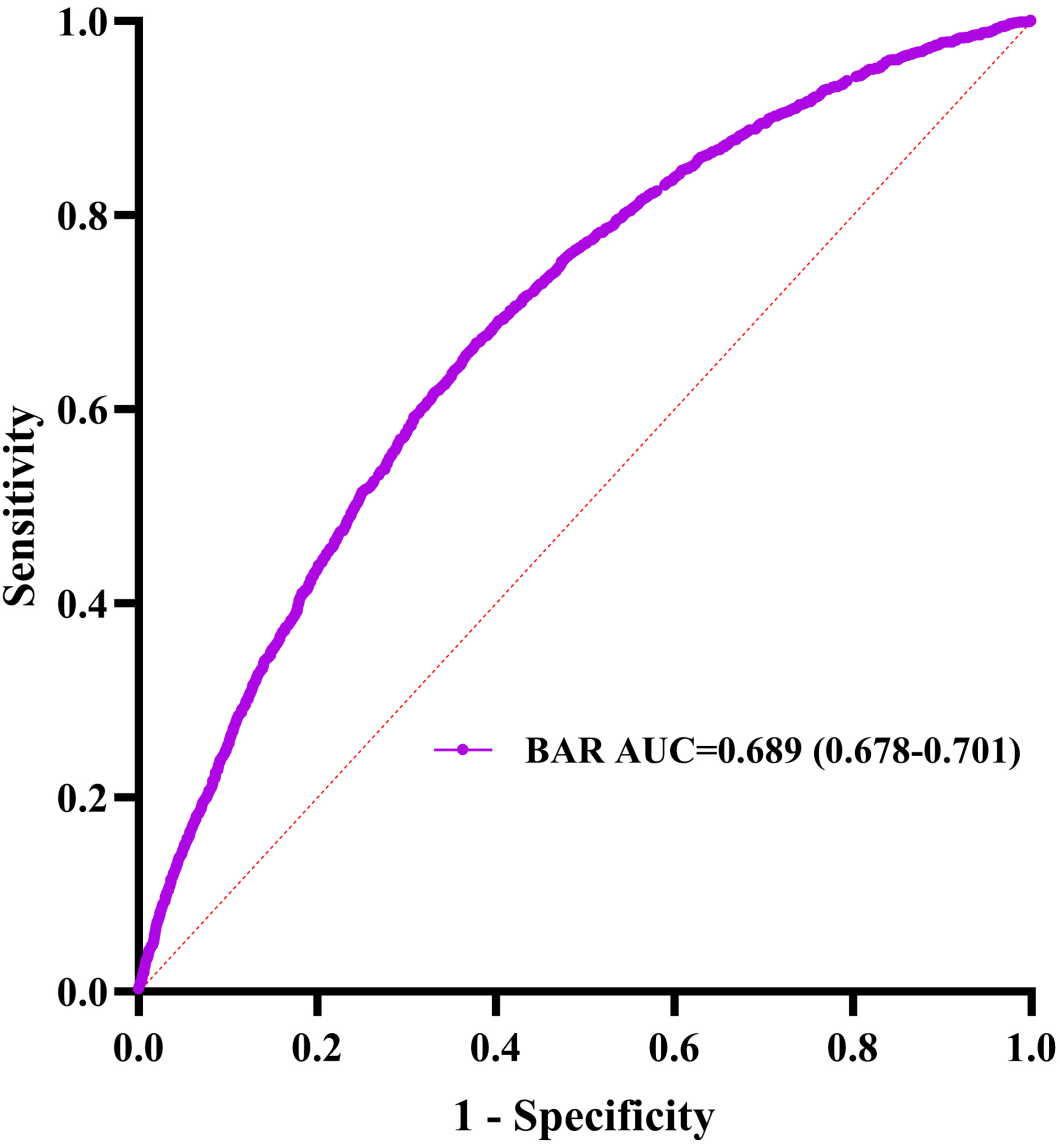
**Blood urea nitrogen receiver operating characteristic curve analysis for in-hospital mortality prediction**. AUC, area under the curve.

The result presented in Table 2, which shows the number of patients at risk during follow-up, stratified by BAR levels. Kaplan-Meier survival analysis demonstrated significantly higher in-hospital survival in patients with BAR ≤8.9 versus BAR >8.9 (HR: 3.15, 95% CI: 2.89–3.44; *p* < 0.001; Fig. 3). The discriminative power of BAR was further validated by a receiver operating characteristics-area under the curve (ROC-AUC) of 0.689 (95% CI: 0.678–0.701; Fig. 4). To verify robustness, sensitivity analysis using a Youden-index-derived cutoff (7.56) revealed consistent predictive trends across alternative thresholds (**Supplementary Fig. 1**, **Supplementary Table 1**). Notably, BAR exhibited a strong positive correlation with serum creatinine (r = 0.732, *p* < 0.001) and a weaker but significant association with white blood cell count (r = 0.145, *p* < 0.001) in atrial fibrillation patients within the ICU cohort (**Supplementary Figs. 2,3**).

**Table 2.** **Number at risk for patients stratified by BAR levels during follow-up**.

Time (days)	Low-BAR (≤8.9)	High-BAR (>8.9)
	N = 8672	N = 4779
10	8032	3910
20	7850	3629
30	7802	3526
40	7774	3493
50	7760	3467
60	7748	3462
70	7746	3456
100	7741	3449

### 3.3 The Nomogram Incorporating BAR Demonstrated Strong Discriminative Ability in Predicting Outcomes

**Fig. 5.**
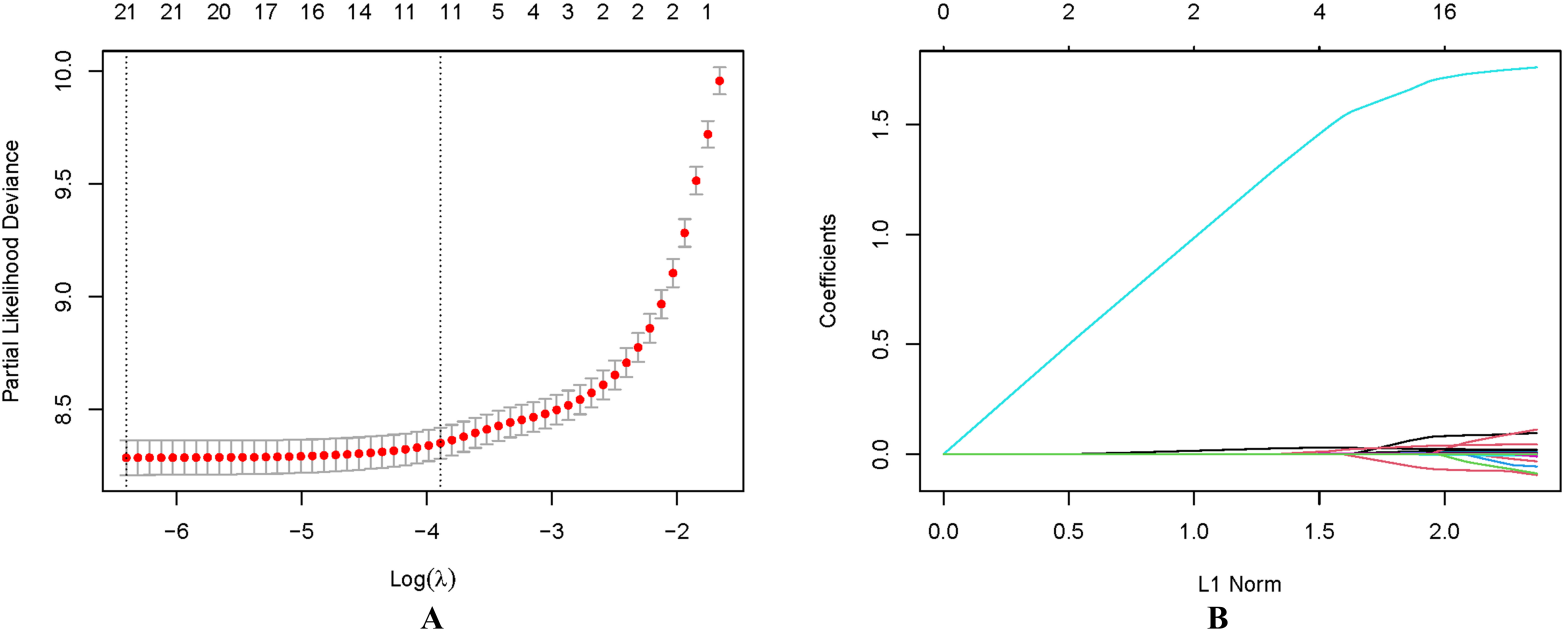
**Finding the important variables associated with hospital death in critically sick AF patients applying the analysis of least absolute shrinkage and selection operator (LASSO) regression**. (A) Using a rigorous cross-validation strategy to optimize the λ parameter of the LASSO regression model; (B) Analyzing the variability patterns of the variable coefficient.

**Fig. 6.**
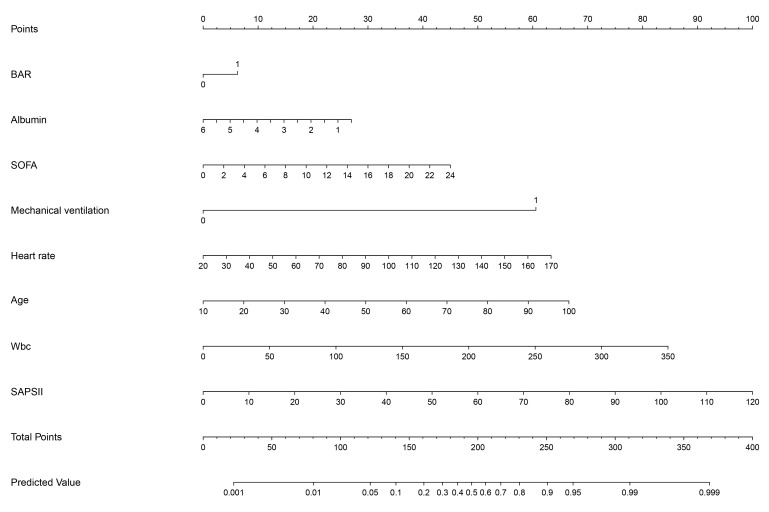
**The nomogram displays the scoring system of the predictive model for in-hospital mortality among critically ill patients with AF**. Wbc, white blood cell count.

**Fig. 7.**
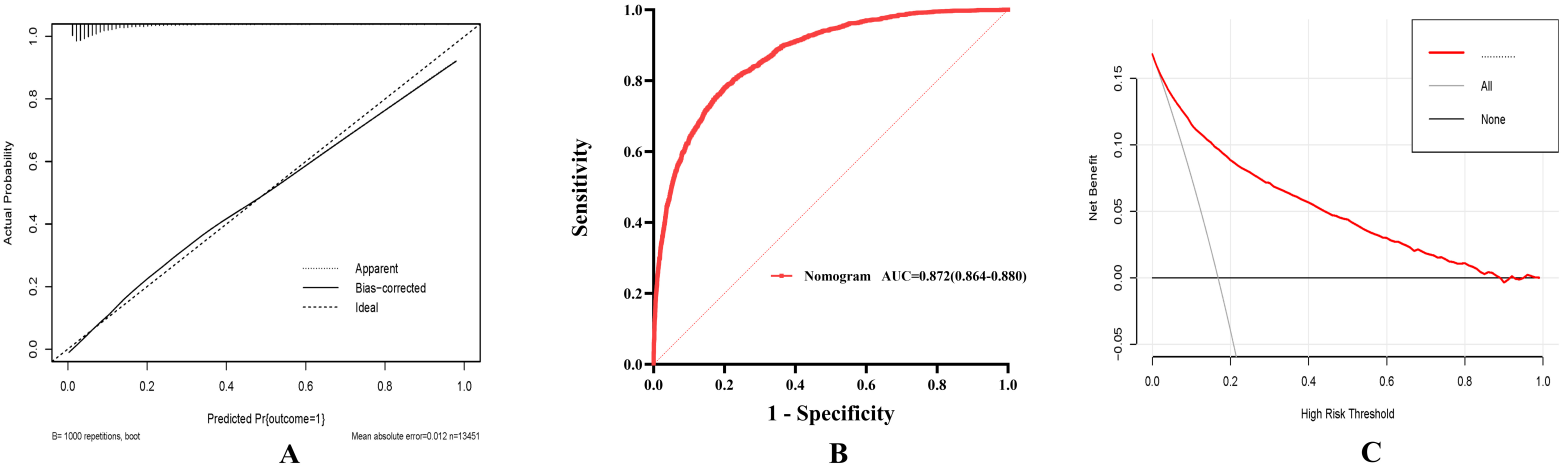
**To determine the clinical utilities of the predictive nomogram**. (A) The in-hospital mortality prediction calibration curve. (B) Blood urea nitrogen and nomogram receiver operating characteristic curve analysis for in-hospital mortality prediction. (C) Predicting in-hospital mortality using the nomogram’s decision curve analysis (DCA).

This study aimed to develop a predictive model for in-hospital mortality in AF patients. Ten risk indicators were initially selected using LASSO regression (Fig. 5). Multivariate logistic regression analysis (Table 3) was then applied to construct a nomogram integrating independent predictors: age (odds ratio, [OR] = 1.030, 95% CI: 1.025–1.035, *p* < 0.001), heart rate (OR = 1.017, 95% CI: 1.014–1.021, *p* < 0.001), white blood cell count (OR = 1.010, 95% CI: 1.005–1.015, *p* < 0.001), albumin level (OR = 0.824, 95% CI: 0.753–0.902, *p* < 0.001), SOFA score (OR = 1.077, 95% CI: 1.054–1.100, *p* < 0.001), SAPS II (OR = 1.034, 95% CI: 1.028–1.040, *p* < 0.001), mechanical ventilation requirement (OR = 11.204, 95% CI: 9.781–12.835, *p* < 0.001), and BAR (OR = 1.284, 95% CI: 1.126–1.464, *p* < 0.001). The nomogram (Fig. 6) visualized cumulative risk stratification, with higher total scores correlating significantly with elevated mortality risk. The calibration curve of the predictive nomogram demonstrated a strong agreement between predicted and actual probabilities, with a mean absolute error of 0.012, indicating high predictive accuracy (Fig. 7A). Furthermore, Fig. 7B shows that the predictive nomogram’s AUC was 0.872 (0.864–0.880), which indicates high predictive performance for in-hospital mortality. Lastly, decision curve analysis (DCA) was used to validate the predictive nomogram’s clinical value, indicating that it might help with clinical decision-making (Fig. 7C).

**Table 3.** **Multivariable logistic regression analysis of LASSO-derived mortality predictors**.

	Univariate		Multivariate	
	OR (95% CI)	*p* value	OR (95% CI)	*p* value
Age	1.025 (1.022–1.028)	<0.001	1.030 (1.025–1.035)	<0.001
Vasopressors	1.112 (1.106–1.118)	<0.001	1.029 (0.896–1.182)	0.686
Mechanical ventilation	12.552 (11.288–13.957)	<0.001	11.204 (9.781–12.835)	<0.001
SOFA	1.248 (1.234–1.263)	<0.001	1.077 (1.054–1.100)	<0.001
OASIA	4.207 (3.826–4.625)	<0.001	0.999 (0.990–1.008)	0.801
SAPS II	1.075 (1.071–1.078)	<0.001	1.034 (1.028–1.040)	<0.001
Heart rate	1.017 (1.014–1.02)	<0.001	1.017 (1.014–1.021)	<0.001
White blood cell	1.028 (1.023–1.033)	<0.001	1.010 (1.005–1.015)	<0.001
Albumin	0.487 (0.455–0.521)	<0.001	0.824 (0.753–0.902)	<0.001
BAR	3.206 (2.922–3.518)	<0.001	1.284 (1.126–1.464)	<0.001

SOFA, sequential organ failure assessment; OASIS, Oxford Acute Severity of Illness Score; ICU, intensive care unit; SAPS II, simplified acute physiology score II; OR, odds ratio.

Multivariable Cox regression analysis (Table 4) demonstrated that the BAR retained significant prognostic value for in-hospital mortality, persisting as an independent predictor across sequentially adjusted models.

**Table 4.** **Independent prognostic factors of in-hospital mortality: Cox regression outcomes**.

Methods		HR (95% CI)	*p* value
For continuous variable, BAR			
	Unadjusted	1.041 (1.038–1.044)	<0.001
	Adjusted for model I	1.039 (1.036–1.042)	<0.001
	Adjusted for model II	1.008 (1.004–1.012)	<0.001
	Adjusted for model III	1.006 (1.002–1.011)	0.004
For categorical variable, BAR			
	Unadjusted	2.850 (2.621–3.099)	<0.001
	Adjusted for model I	1.302 (1.179–1.438)	<0.001
	Adjusted for model II	1.221 (1.102–1.352)	<0.001
	Adjusted for model III	2.577 (2.366–2.808)	<0.001

Model I: Adjusted for age, gender, weight. Model II: Adjusted for age, gender, weight, myocardial infarct, congestive heart failure, diabetes, chronic kidney disease, charlson comorbidity index, mechanical ventilation, vasopressors. Model III: Adjusted for age, gender, weight, myocardial infarct, congestive heart failure, diabetes, chronic kidney disease, charlson comorbidity index, mechanical ventilation, vasopressors, SOFA score, OASIS score, SAPS II score, mean arterial pressure, heart rate, white blood cell, hemoglobin, platelet.

## 4. Discussion

We are the first to examine the usefulness of BAR, a straightforward and practical indicator, in determining the risk of in-hospital death for AF patients admitted to the intensive care unit. We determined that 8.9 was the ideal BAR cut-off value, and patients were categorized into high- and low-BAR groups accordingly. According to our study, participants in the high-BAR group experienced a greater in-hospital death rate and noticeably longer hospital and intensive care unit stays than those in the low-BAR group. This result emphasizes how well BAR predicts the probability of in-hospital death for AF patients admitted to the ICU. Furthermore, this study not only validates the predictive power of the BUN/albumin ratio (BAR) but also demonstrates the superior performance of a nomogram model that integrates BAR with other prognostic factors in predicting ICU mortality among patients with atrial fibrillation. This model exhibits excellent discrimination and calibration performance, coupled with robust clinical utility. Finally, through multivariate Cox regression analysis, we found that both BAR alone and the combined nomogram model consistently predicted in-hospital mortality among ICU-admitted AF patients across different models, providing a novel basis for clinical prognosis assessment.

AF remains the predominant arrhythmia observed in intensive care units (ICUs), paralleling its status as the most prevalent cardiac rhythm disorder globally. Given the elevated mortality risk among critically ill ICU patients compared to general hospital populations, developing a validated prognostic tool tailored for this high-risk cohort is clinically vital. Our study aimed to address this gap by integrating two readily measurable biomarkers—serum albumin and blood urea nitrogen (BUN)—into a mortality prediction model for ICU-admitted AF patients. This framework not only incorporates these biochemical parameters but also synergizes them with established clinical covariates, which we systematically developed and rigorously validated to stratify in-hospital death risk.

The prognostic utility of the BAR has been explored across multiple cardiovascular and renal conditions. In chronic heart failure, Lin *et al*. [14] identified BAR as an independent predictor of 90-day all-cause mortality, while Zhang *et al*. [18] demonstrated its robust association with ventricular aneurysm risk No-ST-segment elevation myocardial infarction, even after multivariable adjustment. Similarly, Sevdımbas *et al*. [19] linked elevated BAR to adverse outcomes in non-ST-elevation myocardial infarction (NSTEMI) cohorts. Beyond cardiac pathologies, Shi *et al*. [20] established BAR as a prognostic marker for 28-day and 1-year mortality in acute kidney injury, and Liu *et al*. [21] further validated its predictive value for long-term mortality in type 2 diabetic nephropathy.

Our analysis demonstrated the robust prognostic value of the BAR for predicting in-hospital mortality in critically ill AF patients. Patients with BAR <8.9 exhibited significantly higher survival rates compared to those with BAR ≥8.9 (HR = 3.15, 95% CI: 2.89–3.44; *p* < 0.001). Utilizing LASSO and multivariate logistic regression, we developed an interpretable risk prediction model incorporating eight independent predictors: BAR, age, heart rate, white blood cell count, albumin, SAPS II, SOFA score, and mechanical ventilation requirement. Advanced age, a well-established prognostic marker for AF [22, 23, 24], emerged as a key variable. Elevated heart rate was independently associated with mortality, aligning with prior evidence indicating higher mortality in AF patients with heart rates >114 bpm versus 90–114 bpm [25]. This observation is further supported by studies linking resting heart rates >81 bpm to increased mortality in AF-associated heart failure [26]. Our study also emphasizes how an elevated white blood cell count predicts mortality risk in AF patients hospitalized to the intensive care unit. Numerous investigations have confirmed the predictive significance of white blood cell count in AF [27, 28, 29], with elevated counts raising the probability of both surgical recurrence and mortality. SAPS II, a tool used to assess disease severity and predict prognosis in critically ill patients, is based on physiological indices and clinical data. Prior research has reported its correlation with mortality risk in AF patients [30], a finding replicated in our study. SOFA score, another scoring system for evaluating the severity and prognosis of critically ill patients by assessing the degree of dysfunction in major organ systems, emerged as a significant risk factor for mortality in our ICU-admitted AF cohort. While SOFA score is well-recognized for its prognostic value in severe patients, its direct association with AF or ICU-admitted AF patients has not been extensively reported. However, studies have shown its value in predicting outcomes in sepsis and AF complicated by sepsis [31, 32, 33], leading us to speculate that higher SOFA scores, indicative of worse organ function and compounded by inflammation, contribute to higher mortality rates among critically ill AF patients in the ICU. Lastly, our nomogram model includes Mechanical ventilation as a critical indicator. Unquestionably, ICU patients requiring mechanical ventilation often present with unstable vital signs and generally have poorer prognoses. This observation is consistent with previous research reporting similar findings [34].

In our investigation, among AF patients admitted to the intensive care unit, BAR was positively connected with the probability of death. Research explicitly connecting BAR to mortality risk in AF patients, especially those hospitalized to the intensive care unit, is still lacking. We hypothesize that the following could be the cause of this: The urea cycle in the liver transforms BUN, a metabolic result of protein digestion and breakdown, into urea, which is then filtered out by the glomerulus. The concentration of BUN indicates the equilibrium between urea production and renal excretion [35]. First, the formation and progression of CVD are significantly influenced by the interaction between the kidneys and heart [36]. BUN has been linked to the prognosis of cardiovascular disease, according to meta-analyses. Furthermore, serum urea nitrogen is associated with neurohormonal activity in addition to reflecting renal function [37]. An increase in urea nitrogen mirrors the cumulative effects of hemodynamic and neurohormonal changes, leading to impaired renal perfusion [38], where neurohormones are integral to the development and prognosis of AF [39]. Additionally, studies have demonstrated that renin-angiotensin system (RAAS) inhibitors can not only prevent the onset of AF but also improve its prognosis [40]. Secondly, numerous studies have confirmed the predictive value of serum urea nitrogen in the occurrence and prognosis of heart failure [41, 42, 43, 44], and AF and heart failure often coexist [45], suggesting that serum urea nitrogen may also contribute to poor prognosis in AF patients through this mechanism. In the setting of hypoalbuminemia, serum albumin, another component of BAR, has been linked in the past to the prognosis of various CVD [46, 47, 48]. Although AF does not directly cause low albumin levels, we speculate that this may be due to the coexistence of AF and heart failure [45], where heart failure itself is a condition of inadequate organ perfusion due to cardiac overload. In the presence of hypoalbuminemia, further loss of fluid in the circulatory system exacerbates the vicious cycle, leading to poor prognosis [14]. Furthermore, any patient in the ICU, an environment prone to infections, malnutrition, liver dysfunction, and renal diseases, is susceptible to further albumin loss, disrupting fluid balance [47, 49]. Therefore, in our study, an elevated BAR reflects the overall condition and mortality risk among ICU-admitted patients with AF. Based on our findings, we believe that BAR and the risk prediction model have significant value in assessing the prevention and improvement of prognosis for AF patients by ICU physicians. However, further research is needed to elucidate the specific mechanisms underlying this indicator, guiding future clinical treatment strategies.

All research findings and conclusions in this retrospective analysis, which was based on the MIMIC-IV database, were taken from the general circumstances and laboratory test results that were obtained within 24 hours after admission. The relationship between continuous BAR trajectories and the outcomes of AF patients admitted to the intensive care unit is not only not adequately examined, but there is also a lack of prospective validation. Additionally, as this study is based on the single-center MIMIC-IV database, we plan to perform external validation in future research using multicenter databases such as the eICU Collaborative Research Database or Chinese critical care datasets. This will help assess the applicability of BAR across different geographic regions and healthcare systems. Limitations in the MIMIC database data, such as the absence of some AF-related scores and stratifications (such as CHA2DS2-VASc score, anticoagulant drug use, and AF type), also add bias to the findings.

## 5. Conclusions

This study demonstrated that there was a higher risk of in-hospital death for AF patients with high BAR who were admitted to the intensive care unit. Furthermore, the nomogram that combined BAR with other pertinent factors showed strong predictive power in estimating the likelihood of in-hospital death. To further validate our findings, more extensive, prospective research is required in the future.

## Availability of Data and Materials

The datasets used during the current study available from the corresponding author on reasonable request.

## Supplementary Material

Supplementary material associated with this article can be found, in the online version, at https://doi.org/10.31083/RCM36596.
