# Supplementary material for: Usefulness of Blood–Urea–Nitrogen to Serum Albumin Ratio for In-hospital Mortality Predictions in Atrial Fibrillation Patients Admitted to the Intensive Care Unit: A Retrospective Analysis From MIMIC-IV Database
Source: Rev Cardiovasc Med. 2025 Jul 29;26(7):36596. doi: 10.31083/RCM36596 (PMC12326415; doi:10.31083/RCM36596)
Supplement: Supplementary file 1 [file 2153-8174-26-7-36596-s1.docx]

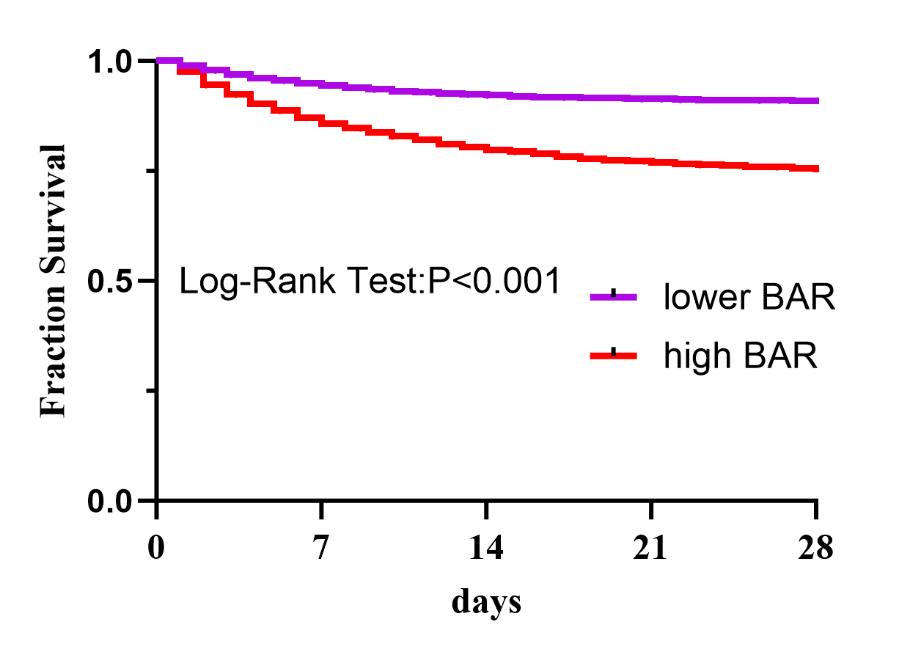


**Supplementary Fig. 1**. **The Kaplan–Meier survival curves were constructed to compare overall survival between high- and low-BAR groups, with the cutoff value of 7.56 determined based on the Youden index.** BAR is the ratio of blood urea nitrogen to serum albumin.


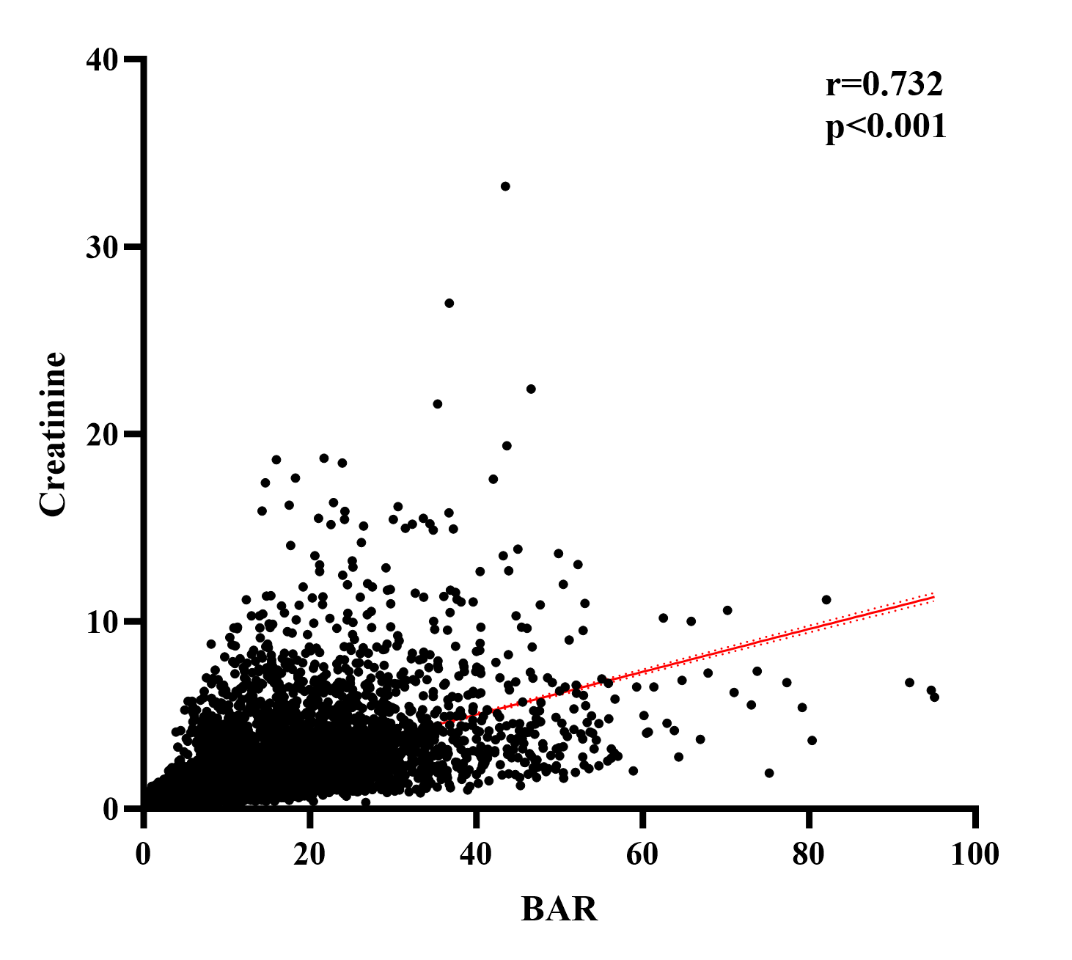


**Supplementary Fig. 2. The correlation between creatinine and BAR.** BAR is the ratio of blood urea nitrogen to serum albumin.


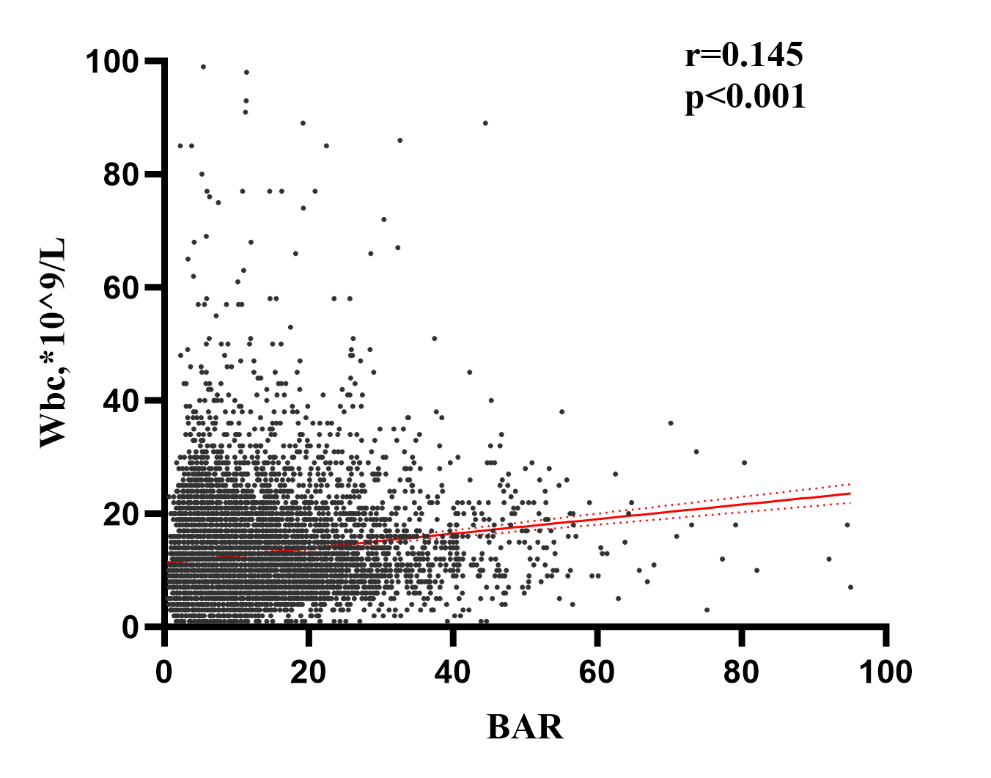


**Supplementary Fig. 3**. **The correlation between wbc and BAR.** BAR is the ratio of blood urea nitrogen to serum albumin.

Supplementary Table 1. Univariate Cox regression analysis for in-hospital mortality.

| Methods | **HR** (95%**CI**) | ***P*** value |
| --- | --- | --- |
| For categorical variable, BAR | | |
| Unadjusted | 2.850(2.621-3.099) | **<0.001** |

**Cutoff value of 7.56 determined is based on the Youden index.** BAR is the ratio of blood urea nitrogen to serum albumin.
